# Cognitive flexibility training intervention among children with autism: a longitudinal study

**DOI:** 10.1186/s41155-017-0069-5

**Published:** 2017-07-25

**Authors:** Cristina de Andrade Varanda, Fernanda Dreux Miranda Fernandes

**Affiliations:** Departamento de Fonoaudiologia, Fisioterapia e Terapia Ocupacional, Universidade de São Paulo–USP-Faculdade de Medicina, Rua Cipotânea, 51, CEP 05360-000 São Paulo, SP Brazil

**Keywords:** Autism spectrum disorders, Cognition, Intervention, Play

## Abstract

Autism is defined by persistent deficits in social communication and social interaction in multiple contexts as well as restricted and repetitive patterns of behavior. There are also reported difficulties in the dynamic activation and modification of cognitive processes in response to changes in tasks’ demands. Such difficulties are believed to be due to poor flexible cognition. This research aimed to assess and intervene in cognitive flexibility in subjects with autism.

Ten subjects diagnosed with autism by psychiatrists, aged 5 years to 13 years and 5 months, were assessed in non-verbal intelligence through Raven’s Progressive Matrices in pretest. They were also assessed in cognitive flexibility through Wisconsin Card Sorting Test (WCST) and in patterns of social interactions, behaviors, and communication through Autism Diagnostic Interview-Revised (ADI-R). An intervention program of 14 to 21 sessions was established to enhance cognitive flexibility. In posttest, they were assessed in WCST and ADI-R.

All measures of cognitive flexibility improved in posttest except for failure to maintain set. Among the measures improved in posttest, perseverative errors and responses improved in posttest with statistical significance as well as categories completed. Total scores on ADI-R were lower in posttest as well as scores on communication abilities.

The qualitative improvement showed by the individuals of this research concerning cognitive flexibility and also patterns of restricted behavior, social interaction, and communication abilities suggests that individuals with autism can benefit from the development of strategies for the enhancement of cognitive flexibility. Nevertheless, more research is suggested with a larger sample among subjects on the autism spectrum.

**Electronic supplementary material:**

The online version of this article (doi:10.1186/s41155-017-0069-5) contains supplementary material, which is available to authorized users.

## Background

Autism is a disorder characterized by alterations present since the age of three and by qualitative impairments in communication and social interaction and by the presence of restricted patterns of interest and behavior. ASDs can be conceptualized as a spectrum disorder with hierarchical levels of severity, as described in DSM-5 (American Psychiatric Association, 2013). In 2010, there were an estimated 52 million cases of ASDs, which means a prevalence of 7.6 per 1000 or one in 132 persons (Baxter, Brugha, Erskine, & Scheurer, 2015). Considering its prevalence and the large scope of affected areas, autism is one of the most disabling developmental disorders (Mehta, Gandal, & Siegel, 2011; Varanda, 2011). The impairments in communication, social interaction, and the use of imagination can be explained through different theories.

One of the theories that explain autism states that executive functions related to planning, working memory, impulse control, inhibition, and mental flexibility are typically impaired in people with neurodevelopmental disorders involving frontal lobe deficits. Such clinical disorders include attention-deficit hyperactivity disorder (ADHD), obsessive-compulsive disorder, Tourette’s syndrome, phenylketonuria, and schizophrenia (Hill, 2004). According to this theory called as executive dysfunction theory, the difficulties in communication and social interaction and, mainly, the restricted patterns of interests and behavior could be explained by inadequate ability for planning, deficient working memory abilities, lack of control and impulse inhibition, and insufficient mental flexibility (Ozonoff, Pennington, & Rogers, 1991; Lopez, Lincoln, Ozonoff, & Lai, 2005).

For the complex characterization of the executive functions, it is necessary to describe its hierarchical structure with the characterizations of its subfunctions (Zelazo & Müller, 2002). Concerning the implication of hierarchical structure of executive functions in performing a task, it is possible to describe different phases along its performance as it happens when attempting to solve a problem during Wisconsin Card Sorting Test (Cunha, Trentini, Argimon, Oliveira, Werlang et al., 2005). At first, there is an intention, after the detection of a pattern, or an elaboration of the representation of the problem to be solved as well as a plan to solve it. After that, it is necessary that such a plan is kept in mind during sufficient time in order to guide the thought action. The action is executed, and the mistakes are then evaluated for a probable correction. The inflexibility that negatively interferes with the execution and success of the action can occur at any described phase. In the specific situation of Wisconsin Card Sorting Test (Cunha et al., 2005), the inflexibility may take place when a change in the sorting rule occurs and the subject perseveres in the previous rule because a new plan was not formed or was formed but was not executed. The executive function analysis based on this model allows the detection of where there is a break in the process of problem solving. Executive functions can be understood through a problem-solving framework.

Flexible cognition or cognitive flexibility “entails the dynamic activation and modification of cognitive processes in response to changing task demands” (Déak, 2003, p. 275). When demands and task context change, the cognitive system can adapt, shift attentional focus, select information to guide and choose necessary responses, form plans, and generate new activation to feed back into the system. If those processes result in well-adapted representations and actions, it is possible to state that those processes succeeded due to flexible cognition or cognitive flexibility.

Cognitive flexibility is involved in systems of actions including the use of tools, social interaction, spatial navigation, planning, and creative thinking. Besides all those, cognitive flexibility has a fundamental role in the development and well-succeeded use of language, once for acquiring language, one has to have a rich representational system at hand as well as flexible means to decide among the linguistic representations available (Déak, 2003).

The behavior of people with autism that could be explained by the executive dysfunction theory includes the need for routine, strong preference for repetitive behavior, lack of impulse control, difficulty in initiating new actions, and difficulty of shifting from one task to another (Hill, 2004; Hill & Frith, 2003; Hughes, Russell, & Robbins, 1994). However, Rajendran and Mitchell (2007) cite some authors who, after having evaluated individuals with autism and individuals with attention-deficit hyperactivity disorder and Tourette’s syndrome, concluded that individuals with autism presented a specific deficit in cognitive flexibility, while inhibitory control, for instance, seems to be less affected than in other disorders. Although deficits in executive functions can be present in various clinical disorders, individuals with autism has shown significant deficits in EF domains, especially in planning and cognitive flexibility, when compared to individuals with attention-deficit hyperactivity disorder, for instance (Geurst, Verté, Oosterlaan, Roeyers, & Sergeant, 2004). In fact, difficulties in cognitive flexibility in autism are related to deficits in the theory of mind, communication skills, and maladaptive behaviors (Memari, Ziaee, Shayestehfar, Ghanouni, Mansournia et al., 2013) which are associated with quality of life (de Vries & Geurts, 2015).

Varanda (2011) did a research to assess ten subjects diagnosed with autism in syntactic awareness, central coherence, non-verbal intelligence, social and communicative development, and patterns of behavior and interests to verify the probable relationship among these variables and to detect probable linguistic profiles related to syntactic awareness. In a first study, it was verified that some abilities such as self-monitoring and self-regulation should be developed or refined in order to allow a better performance in syntactic tasks. Syntactic awareness is a metalinguistic task and requires resources from the central executive system such as self-monitoring as self-regulation to be accomplished successfully. When assessing profiles of communication and social interactions, Varanda (2011) ascertained the presence of failure in social interaction attempts and social response adequacy, indicating a probable primary deficit, which is joint attention deficit. Joint attention refers to social cognition and a variety of behaviors such as following attention (imitation, social reference) and drawing attention such as pointing, language, protodeclarative, and protoimperative gestures (Stahl & Pry, 2004). Difficulties in the fast shifting of attentional focus can contribute to an increase in joint attention failure. According to Stahl and Pry (2004), the need for attention in these abilities seem to be part of a triad, demanding flexible transition between attention to oneself, to some object or event, and to someone else. Therefore, cognitive flexibility would be directly related to failure in joint attention. These two studies had the aim to test the previous hypothesis that there would be a relationship between syntactic awareness and central coherence, non-verbal intelligence, social and communicative development, as well as development of behaviors and interests and to test the hypothesis that there would be varied linguistic profiles concerning syntax among subjects with autism. However, only in her third study (Varanda, 2011) that was based on analyses made on case reports, relying on information given by the parents of the individuals with autism in an interview, it was possible to formulate a hypothesis instead of corroborating hypotheses previously thought as in the other two studies. The two studies developed in laboratory testing the subjects under controlled conditions together with the complementary findings of the third study offered high ecological validity. Therefore, based on the reports provided by the parents of the individuals with autism, it was possible to infer that cognitive flexibility or the lack of it is present in all the difficulties showed by the subjects not only in communication but also in social interaction and behavioral patterns. According to this idea, cognitive flexibility cannot be understood as an isolated ability but as a unified account which can be considered a property of the cognitive system. Cognitive flexibility requires the interaction of several mechanisms (e.g., attention shifting, conflict monitoring, and perception) that respond to specific environmental demands (such as rule changes) in order to achieve flexible behavior (such as solving a problem in a new way) (Ionescu, 2012). Children with autism show developmental core deficits in joint attention which is a prelinguistic communicative ability and also in play (Kasari, Chang, & Patterson, 2013). Playing in autism is characterized by reduced frequency, complexity, novelty, and spontaneity (Kasari, Huynh, & Gulsrud, 2011). In fact, playing becomes an important focus of intervention in autism once developmental indicators of difficulties in autism are a relative absence of creativity and fantasy in the processes of thought relating strongly with social, cognitive, symbolic, and linguistic development. Playing favors abstractions, generalizations, and transitions of meanings (Amato et al., 2014). When considering cognitive flexibility as a property of the cognitive system requiring the interaction of attention shifting, conflict monitoring, and perception, it is possible to argue that sameness in play showed by individuals with autism is related to the lack of cognitive flexibility. However and although play is considered an important part of development and deficits in playing can be detected among children with autism, those children lack play experiences with others decreasing their exposure and development in social play (Kasari et al., 2013).

Regarding therapeutical efforts to improve cognitive flexibility among individuals with autism, the effects of direct EF interventions in ASD have presented mixed results, either positive or null (de Vries, Prins, Schmand, & Geurts, 2015). Nevertheless, once ASD has been lately understood as a disorder with a neurobiological explanation, the idea of remediating the core brain deficits involved in social and non-social cognitive dysfunction in ASD has been discussed (Eack, Bahorik, Hogarty, Greenwald, Litschge et al., 2013). Cognitive rehabilitation can vary largely (Eack et al., 2013), but cognitive flexibility which can be associated with multiple abilities can offer a promising alternative. When focusing on the improvement of cognitive flexibility among children with autism, researchers and therapists must consider not only the deficits related to autism but also the fact that the choice of strategies for improving flexible cognition must take the level of children’s development into account. Bergen (2016) compares two broad approaches for the minimization of ASD difficulties. One of them is based on the theory of behavioral modification, and the other one is based on the theory of human development which focuses on the promotion of typical play as a mediator of autistic behavior. The author mentions the proposal that emotional organization is a primary task of children and mentions the typical stages of such emotional organization, mentioning the recommendation of Greenspan (as cited in Bergen, 2016). The mentioned developmental stages can be stimulated through an approach based on playing called *Floortime*. Bergen (2016) stated this method is similar to guided play in which there is the involvement of adults and children in complementary interactions in playful activities. Guided play is different from free play once incorporates adult-scaffolded learning objectives remaining child-directed (Weisberg, Hirsh-Pasek, & Golinkoff, 2013). Another issue concerning the focus of cognitive interventions is that it should have a comprehensive approach considering not only the cognitive deficits but also and mainly social-cognitive ones (Eack et al., 2013).

Cognitive flexibility has been pointed as an ability which allows people to be successful in swifting their attentional focus in complex tasks such as social interaction situations, for instance, and deficits in this ability can harm other abilities in autism as verified by Varanda (2011). Therefore, the suggestion of using play and games for the improvement of cognitive flexibility, planning ability, and flexible use of language among children and adolescents with autism can minimize the negative impact that the lack of behavioral repertoire presented by individuals with autism for accomplishing daily tasks at home and school. In fact, cognitive flexibility seems to be a core ability for the development of a satisfactory pattern of social interaction which allows these individuals to actively take part in life in society. Faja, Dawson, Sullivan, Meltzoff, and Estes et al. (2016) argue that executive dysfunction contributes to playing deficits in ASD. They state that early executive function skills may be critical in order for verbal children with autism to develop play.

This research aimed to assess the present developmental level of children and adolescents with autism in communication, social interaction, and patterns of behavior and interests, in non-verbal intelligence and cognitive flexibility. Besides, it proposed the use of playing activities and games for the development and enhancement of cognitive flexibility and planning. After intervention, gains in communication, social interaction, and patterns of behavior and interests and cognitive flexibility were checked.

## Methods

### Participants

The participants were ten children and adolescents, with ages ranging from 5 years and 5 months to 13 years and 5 months, in the beginning of the study, enrolled in language therapy processes in the Speech and Language Pathology in Autism Spectrum Disorders Research Laboratory of the School of Medicine, University of São Paulo, Brazil. Their psychiatric diagnosis was determined by psychiatrists and neurologists according to the criteria proposed by the DSM-IV TR (APA, 1995). The inclusion criteria were the ability to cooperate and participate by answering orally to the tests and to attend the speech-language therapy sessions. All the participants should have oral language. Before the intervention of the present study, in 2012 and 2013, the subjects presented a general mean of communicative acts per minute of 7.88 and a general mean of occupation of the communicative space of 76.6%. Communicative acts per minute and occupation of the communicative space are pragmatic aspects of the subjects’ communication as proposed by Fernandes (2000). These means are calculated based on the analyzed corpus of videotaped speech of the subjects. This was a longitudinal study which lasted 3 years.

### Instruments

The participants were tested in non-verbal intelligence through Raven’s Progressive Matrices in its Portuguese edition (Angelini, Alves, Custódio, Duarte, & Duarte, 1999). This test measures the ability to deduce relationships within geometric patterns or among figural elements contained in a matrix. It minimizes the influences of verbal communication and past experience (Robertson, 2010).

For assessing communication, social interaction, and patterns of behavior and interests, the current behavior algorithm of ADI-R was used (Rutter, Le Couteur, & Lord, 2003) which is an extensive semi-structured interview including three domains of autism symptoms (social, communication, and repetitive behavior). The more difficulties the individual presents, the higher are the scores in the test.

They were tested in cognitive flexibility through WCST (Cunha et al., 2005; Dawson & Guare, 2010; Kaland, Smith, & Mortensen, 2008) by the first author, that is, an experienced professional. The WCST is a problem-solving, neuropsychological test, which requires the respondent to understand the logical principles of the proposed problem. The test proceeds through a number of shifts in set (sorting principles) that varies along three dimensions (color, form, and number). Successful performance on the WCST requires the participant first to determine the correct sorting principle and to maintain this sorting principle or set. Scores were based on number of trials administered, total number of correct responses, number of errors, number of perseverative responses, number of perseverative errors, number of non-perseverative errors, number of categories completed, number of trials to complete the first category, conceptual level responses, failure to maintain set, and learning to learn.

The categories *number of trials administered*, *total number of correct answers*, *total number of errors*, *number of perseverative responses*, *number of perseverative errors*, *number of categories completed*, *number of trials to complete the first category*, and *failure to maintain set* were considered for analyzing gains related to cognitive flexibility in the present study.

### Procedure

The research was proceeded with the consent of the Ethics Committee for the Analysis of Research Projects of the School of Medicine of Universidade de São Paulo (USP) under number 408/12. The adults responsible for the children signed the approved informed consent form. The individuals were tested in 2012 and 2013. The intervention took place in 2014, and they were assessed in the same measures in posttest in 2015. The children and adolescents were diagnosed on the autism spectrum by psychiatrists and attended language therapy sessions in the Autism Spectrum Disorders Speech and Language Research Laboratory of the School of Medicine, University of São Paulo, Brazil.

All the ten children were assessed in non-verbal intelligence through Raven, in communication, social interaction, and patterns of restricted interests and repetitive behavior through ADI-R from the verbal algorithm and in cognitive flexibility through WSCT.

After intervention, all the subjects were reassessed in communication, social interaction, and patterns of restricted interests and repetitive behavior through ADI-R and in cognitive flexibility through WSCT.

### Intervention

There were a total of 21 sessions. During their weekly language therapy sessions, 20-min play activities were performed to improve cognitive flexibility (Carvalho & Nomura, 2004; Jolliffe & Baron-Cohen, 1999; Oaklander, 1980). After every two sessions targeting specific abilities related to cognitive flexibility, such abilities were assessed in the beginning of the following session with a test (Galifret-Granjon & Santucci, 1981; Kulaif, 2005; Lezak, 1995; Montiel & Seabra, 2009; Seabra, Assef, & Cozza, 2009). The list of activities used in therapeutic sessions and pictures illustrating them during intervention with the respective tests used for the verification of gains in the abilities focused in previous sessions are presented in the Additional file 1. These activities were proposed because they were easy to be included in the activities developed during the language intervention sessions by the speech-language pathologist.

### Statistical analysis plan

This was a quasi-experimental study with a one-group pretest-posttest design.

Measures of central tendency of ADI-R and WCST scores in pre- and posttest were obtained in order to describe the whole group (ten subjects) in both conditions. The percentiles of Raven’s test were used for describing the intellectual profile of the subjects. The non-parametric Wilcoxon matched signed-rank test was used for verifying any difference in the mean of pre- and posttest measures in WCST items and ADI-R.

## Results

Among the ten subjects, only one of them was a girl. In the beginning of the research, their ages varied from 5 years and 5 months to 13 years and 5 months. Their mean age was of 8y2m.

Regarding non-verbal intelligence, only 30% of the subjects were found to be in the intellectually deficient range according to the results of Raven. Table 1 shows the age and percentiles of Raven’s test of all the ten subjects.

**Table 1:** Age and Raven’s percentiles of the ten subjects

Subjects	Age	Raven’s percentiles
1	5.5	95
2	6.8	10
3	6.2	99
4	8.0	99
5	9.2	10
6	5.5	99
7	7.9	60
8	10.4	1
9	9.2	60
10	13.5	60

Regarding the assessment of communication, social interaction, patterns of restricted interests and stereotyped behavior, as well as cognitive flexibility, Tables 2 and 3 present these results.

**Table 2:** Measures of central tendency in ADI-R of the ten subjects in pretest and posttest

	Mean		Median		SD		Minimum		Maximum	
*N* = 10	Pre	Post	Pre	Post	Pre	Post	Pre	Post	Pre	Post
Social interaction	11.1	8.9	10.5	10	5.7	4.6	3	2	20	16
Communication	13.1	9.2	13	10	4.2	4	6	3	21	15
Restricted interests/behavior	6.2	6	6	6	3.1	2.3	1	1	10	9
Total	30.4	24.1	28	26.5	9.9	9.1	13	8	50	40

**Table 3:** Measures of central tendency in Wisconsin Card Sorting Test—WCST of the ten subjects in pretest and posttest

	Mean		SD		Minimum		Maximum	
*N* = 10	Pre	Post	Pre	Post	Pre	Post	Pre	Post
Number of trials administered	123.3	117.7	14.9	19.4	81	68	128	128
Total number of correct answers	60.4	68.1	25.8	19.9	32	35	102	94
Total number of errors	62.9	49.7	31.4	29.4	11	4	96	93
Number of perseverative responses	58.6	31.9	46.9	30.3	6	1	127	88
Number of perseverative errors	46.3	26.1	34.5	23.8	6	1	95	71
Number of categories completed	2.1	3.8	2.4	2.2	0	0	6	6
Number of trials to complete the 1st category	61	34.5	58.7	37.2	10	10	129	128
Failure to maintain set	1.2	1.6	1.5	1.7	0	0	4	4

Concerning the measures of cognitive flexibility assessed with WCST, only the test items *perseverative errors*, *perseverative responses*, and *categories completed* showed significant differences between pre- and posttest. An analysis with a Wilcoxon matched signed-rank test demonstrated that perseverative errors and perseverative responses in pretest accounted for most of the errors and categories completed performance improved in posttest as shown in Table 4.

**Table 4:** Differences in perseverative errors, responses, and categories completed comparing pre- and posttest

Test items	Mean	SD	Median	*p* value
Perseverative errors (pre)	46.3	34.5	35	0.028
Perseverative errors (pos)	26.1	23.8	18	
Perseverative responses (pre)	58.6	46.9	38.5	0.028
Perseverative responses (pos)	31.9	30.3	19.5	
Categories completed (pre)	2.1	2.4	1.5	0.049
Categories completed (pos)	3.8	2.2	4	

The general performance on ADI-R improved in posttest (lower punctuation) with a trend towards statistical significance on Wilcoxon (*p* = 0.051). The “communication” domain of ADI-R improved in posttest showing statistical significance on Wilcoxon (*p* = 0.033).

## Discussion

Although the subjects were not a cognitively homogenous group, in which only 30% of them accounted for intellectual deficiency, the authors decided to keep those individuals in the study once one of the aims of the research was to verify qualitative gains in communication, interaction, and patterns of behavior and interests as well as cognitive flexibility within this specific group regardless the possibility of generalization of results. All measures in Wisconsin Card Sorting Test were improved in posttest, except for failure to maintain set (FMS). This can be explained by the fact that in solving WCST, one has to maintain attention and not only think flexibly. FMS is calculated in the WCST by counting the number of times an individual fails to sort cards by the sorting rule after it can be determined that he or she has acquired the rule. When individuals fail to maintain set, they incorrectly change their sorting strategy before change is appropriate. This is why FMS can be considered a measure of distractability rather than cognitive flexibility (Figueroa & Youmans, 2013).

WCST may not have good test-retest reliability due to practicing effects (once the person understands the categorization behind the test, it is very likely he/she will remember it in the next administrations). Ozonoff (1995) addressed this issue in a study in which she investigated the test-retest reliability of the standardly administered WCST over a 2′/2-year interval in two clinical samples, one with autism and the other with learning disabilities. The results of her study suggested that the traditional WCST is a highly reliable instrument for use with both autistic and learning-disabled individuals over time. The difference between Ozonoff’s study and studies with participants with normal patterns of development is that in her study, the participants had clinical diagnoses, and participants without disabilities may be more likely to figure out WCST’s “tricks” during initial testing, again potentially increasing scores at time 2 and contributing to lower test-retest coefficients. The length of the test-retest interval was also addressed in her study when she compared her results and those of Heaton, Chelune, Talley, Kay, and Curtiss’ (1993) stating that the generalizability coefficients obtained in her study are substantially higher than the values reported by Heaton et al.’s (1993; rs between .37and .72) for normally developing participants of similar age. In her study, the length of the test-retest interval was considerably greater (2′/2 years) than in Heaton et al.’s study (1 month). In the present study, the mean of test-retest interval was of 2 years and 4 months which is similar to that in Ozonoff’s study.

Perseverative errors and responses were significantly improved after intervention. These are categories assumed to be impaired in autism which interferes negatively with flexible cognition necessary for adapting to different demands in social situations as well as cognitive tasks. They also improved in communicative abilities. This leads to the conclusion that regardless their intellectual abilities, they could improve specific cognitive abilities involved in autism, which can reinforce the idea that cognitive flexibility is a core ability for the development of other important skills for interacting. It is important to mention that the intervention proposed was based on the presumption that cognitive flexibility is not an isolated ability but entails a set of other abilities such as social interaction, communicative abilities, and symbolic play. This is why the intervention consisted of a more comprehensive set of skills all of them related to flexible cognition, such as inference, planning, and spatial orientation.

It is important to say that the participants were also attending language therapy sessions along the present study which contributes to improve social interaction patterns, communicative abilities, and symbolic play. After the end of this research, participants presented a general mean of communicative acts per minute of 8.26 and a general mean of occupation of the communicative space of 73.3%. Differently from the perspective of the subjects’ parents, their level of communication was kept stable without any further significant gains. On the one hand, these results pose the question whether parent report as given in ADI-R is less accurate than direct measures of assessment because parents tend to over-estimate their child’s language, mainly comprehension skills (Charman, 2004). On the other hand, these results do not follow the significant gains obtained in perseverative responses and errors suggesting that these abilities are not necessarily involved in communicative abilities. Therefore, although cognitive flexibility is not an isolated ability entailing a set of other abilities, the results obtained regarding perseverative responses and errors shall not be involved in gains in communicative abilities. The significant gains obtained in perseverative errors and responses are therefore related to poor flexibility but not necessarily to occupation of the communicative space and communicative acts.

## Conclusions

These results suggest that in tasks that require sustained attention, FMS is a measure of distractibility considering the specific profiles.

Once this was a one-group pretest-posttest design, there was only one group selected under observation with a careful measurement being done before applying the experimental treatment and measuring after, external validity cannot be expected. There are limitations widely known in this kind of design such as random events outside the experiment or participants that may affect the measurement, change within the participants which may affect the measurement, and even the testing itself which can affect the participants. Nevertheless, quasi-experimental designs can be an approachable option for populations such as children with autism whose cognitive abilities and availability to participate and to engage in testing and activities are variable. In favor of this argument, the results showed significant difference in posttest supporting internal validity.

Although there may be considerable differences between the profiles of subjects diagnosed on the autism spectrum in this study, the qualitative improvement showed by them concerning flexible cognition and also patterns of restricted behavior and social interaction suggests that individuals with autism in language therapy can benefit from programs to develop and enhance cognitive flexibility which entails a cognitive operation mediated by the prefrontal cortex and that interferes with the individual’s responses which are commonly perseverative.

The level of communicative abilities (communicative acts and occupation of the communicative space) were kept stable comparing the moment before the beginning of the research and after its end. These results are opposed to what the participants’ parents reported in ADI-R. Therefore, although cognitive flexibility is not an isolated ability entailing a set of other abilities, the results obtained regarding perseverative responses and errors shall not be involved in gains in communicative abilities being related to poor flexibility but not necessarily to occupation of the communicative space and communicative acts. These findings suggest that the gains in perseverative errors and responses might not be involved in communicative ability development. Besides that, one-group pretest-posttest design makes it difficult to draw causal conclusions in a longitudinal study once the subjects are exposed to numerous variables that can affect the final assessment.

Nevertheless, the research showed that children with autism under the study’s conditions can benefit from this model of intervention. However, more research under an experimental design is suggested with a larger sample among subjects on the autism spectrum that can be generalized to larger populations of people with autism.

## Additional files

Additional file 1: List of activities used in therapeutic sessions during intervention. (DOCX 675 kb)
