# Supplementary material for: Cognitive flexibility training intervention among children with autism: a longitudinal study
Source: Psicol Reflex Crit. 2017 Jul 25;30:15. doi: 10.1186/s41155-017-0069-5 (PMC6974343; doi:10.1186/s41155-017-0069-5)
Supplement: Supplementary file 1 — List of activities used in therapeutic sessions during intervention. (DOCX 675 kb) [file 41155_2017_69_MOESM1_ESM.docx]

Additional file 1

*List of activities used in therapeutic sessions during intervention*

| **Session** | **Activities names** | **Material type** | **Time** | **Developmental/therapeutic goals** | **Activities used** |
| --- | --- | --- | --- | --- | --- |
| 1 | Tower of Hanoi | digital | 20’ | Cognitive flexibility and plannning | 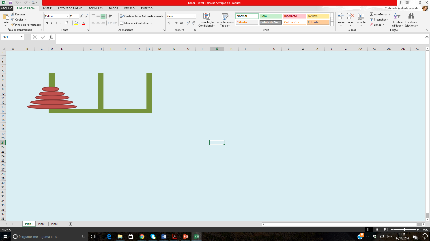 |
| 2 | Tower of Hanoi | concrete | 20’ | Cognitive flexibility and plannning | 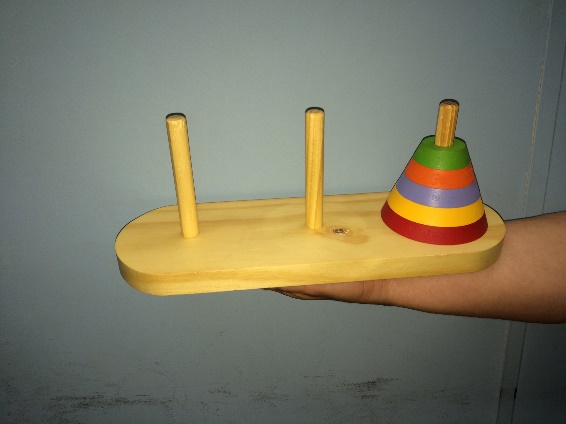 |
| 3 | Tower of Hanoi test (Lezak, 1995) | concrete | 10’ | Cognitive flexibility and plannning | TEST |
|  | *“Mastermind” game* | concrete | 20’ | Cognitive flexibility. plannning and inhibitory control | 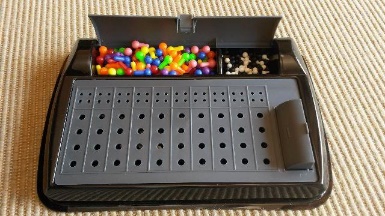 |
| 4 | “Tchuka” game | concrete | 20’ | Cognitive flexibility and plannning | 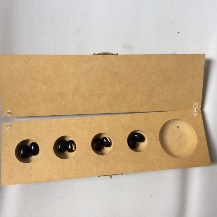 |
| 5 | The trail making test (Seabra, Assef & Cozza, 2009) | paper | 10’ | Cognitive flexibility | TEST |
|  | “Stroop” game |  | 20’ | Cognitive flexibility. plannning and inhibitory control | 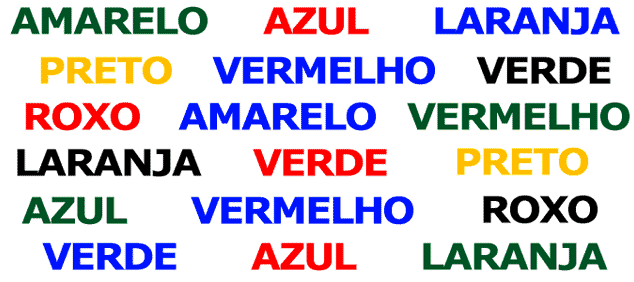 |
| 6 | “Who. when and where” game | concrete | 20’ | Cognitive flexibility | 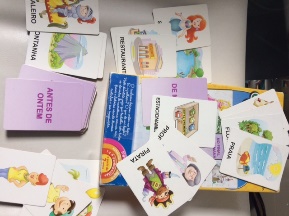 |
| 7 | “Tower of Hanoi” test (Lezak, 1995) | concrete | 10’ | Cognitive flexibility and plannning | TEST |
|  | “Lince” game | concrete | 20’ | Inhibitory control and attention | 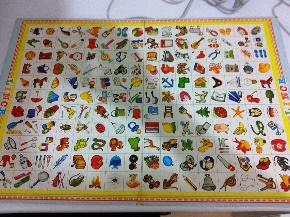 |
| 8 | “Puzzle sticks” | concrete | 20’ | Cognitive flexibility, planning and spatial orientation | 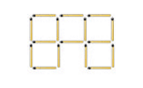 |
| 9 | Attention by cancelling test (Montiel & Seabra, 2009) | paper | 10’ | Inhibitory control and attention | TEST |
|  | Mirror image imitation of positioning toy buildings | concrete | 20’ | Cognitive flexibility and spatial orientation | 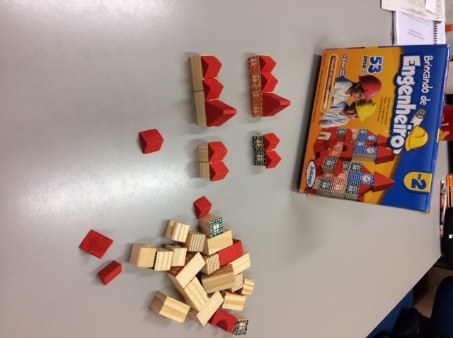 |
| 10 | “Challenge” game | concrete | 20’ | Cognitive flexibility, planning and spatial orientation | 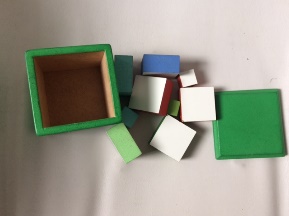 |
| 11 | Constructive praxia (Carvalho & Nomura, 2004)  Cognitive training – From the 1st to the 5th phases | concrete | 20’ | Cognitive flexibility, planning and spatial orientation | 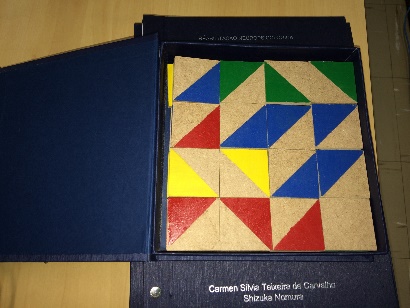 |
| 12 | Constructive praxia (Carvalho & Nomura, 2004)  Cognitive training – From the 6th to the 11th phases | concrete | 20’ | Cognitive flexibility, planning and spatial orientation | 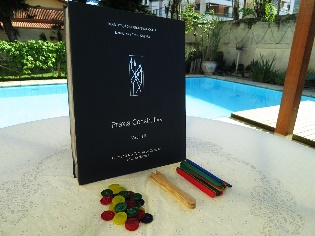 |
| 13 | Mirror image imitation of dolls movements | concrete | 20’ | Cognitive flexibility and spatial orientation | 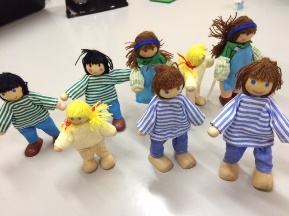 |
| 14 | Right-left orientation test (Galifret-Granjon & Santucci, 1981) | paper | 10’ | Spatial orientation | TEST |
|  | Dramatic play based on story reading (Oaklander, 1980) | concrete | 20’ | Cognitive flexibility, theory of mind and central coherence | 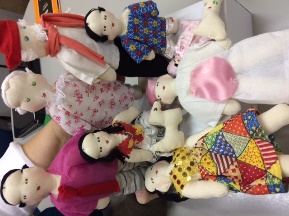  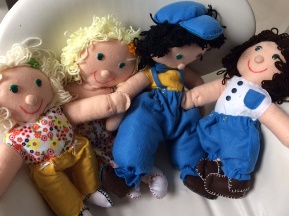 |
| 15 | Dramatic play based on story reading (Oaklander, 1980) | concrete | 20’ | Cognitive flexibility, theory of mind and central coherence |  |
| 16 | Dramatic play based on story reading (Oaklander, 1980) | concrete | 20’ | Cognitive flexibility, theory of mind and central coherence |  |
| 17 | Stories improvisation when dealing with conflicts (Oaklander, 1980) | concrete | 20’ | Cognitive flexibility, theory of mind and central coherence | 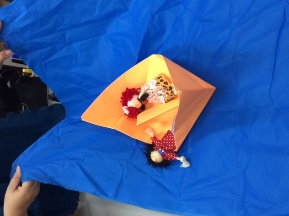 |
| 18 | Colors and Words Modified Stroop Test (Kulaif, 2005) | paper | 10’ | Inhibitory control | TEST |
|  | Stories improvisation when dealing with conflicts (Oaklander, 1980) | concrete | 20’ | Cognitive flexibility, theory of mind and central coherence | 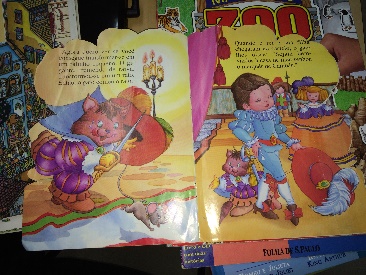 |
| 19 | Local Coherence Inference Task (Jolliffe & Baron-Cohen, 1999) | paper | 10’ | Theory of mind, central coherence and cognitive flexibility | TEST |
| 20 | Instructional training (Carvalho & Nomura, 2004) | paper | 20’ | Cognitive flexibility, planning and inhibitory control | 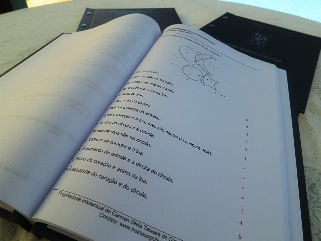 |
| 21 | Instructional training (Carvalho & Nomura, 2004) | paper | 20’ | Cognitive flexibility, planning and inhibitory control | 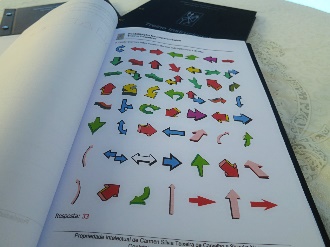 |
